# Long Noncoding RNA *EZR-AS1* Regulates the Proliferation, Migration, and Apoptosis of Human Venous Endothelial Cells via SMYD3

**DOI:** 10.1155/2020/6840234

**Published:** 2020-05-22

**Authors:** Ganhua You, Xiangshu Long, Fang Song, Jing Huang, Maobo Tian, Yan Xiao, Shiyan Deng, Qiang Wu

**Affiliations:** ^1^Guizhou University School of Medicine, No. 2708 South Section of Huaxi Avenue, Guiyang 550025, China; ^2^Guizhou Institute for Food and Drug Control, No. 142 Shibei Road, Guiyang 550004, China; ^3^Department of Cardiology, Guizhou Provincial People's Hospital, People's Hospital of Guizhou University, No. 83 Zhongshan East Road, Guiyang 550002, China

## Abstract

Numerous studies have shown that long noncoding RNAs (lncRNAs) play essential roles in the development and progression of human cardiovascular diseases. However, whether lncRNA ezrin antisense RNA 1 (*EZR-AS1*) is associated with the progression of coronary heart disease (CHD) remains unclear. Accordingly, the aim of the present study was to evaluate the role of lncRNA *EZR-AS1* in patients with CHD and in human venous endothelial cells (HUVECs). The findings revealed that lncRNA *EZR-AS1* was highly expressed in the peripheral blood of patients with CHD. *In vitro* experiments showed that the overexpression of *EZR-AS1* could enhance proliferation, migration, and apoptosis by upregulating the expression of EZR in HUVECs; downregulation of lncRNA *EZR-AS1* resulted in the opposite effect. lncRNA *EZR-AS1* was also found to regulate SET and MYND domain-containing protein 3 (SMYD3), a histone H3 lysine 4-specific methyltransferase, which subsequently mediated EZR transcription. Collectively, these results demonstrate that lncRNA *EZR-AS1* plays an important role in HUVECs function via SMYD3 signaling.

## 1. Introduction

As a result of economic development and an aging population, the risk of developing cardiovascular disease has gradually increased [1, 2]. Coronary heart disease (CHD) is a common, high rate of mortality disease with global influence [3, 4], and it is therefore necessary to optimize treatment and prevention strategies.

Long noncoding RNAs (lncRNAs) are a class of RNA molecules > 200 nucleotides in length, which do not encode proteins [5, 6]. lncRNAs regulate gene expression through epigenetic, transcriptional, and post-transcriptional mechanisms. Recent studies have shown that lncRNAs are also involved in the development and progression of various cardiovascular diseases [7–11], including heart failure, myocardial hypertrophy, heart metabolic disease, myocardial infarction, and atherosclerosis (AS).

Ezrin (EZR) is a member of the ezrin-radixin-moesin (ERM) family of cytoskeletal proteins, which connects the actin cytoskeleton to the plasma membrane. EZR plays a vital role in numerous processes associated with normal cell growth, including adhesion, cell polarity, and migration [12, 13]. SET and MYND domain-containing protein 3 (SMYD3) is a member of the SET and MYND domain (SMYD) family. Studies have shown that SMYD3 interacts with the specific region of trimethylation of histone H3 lysine 4- (H3K4Me3-) modified histone tails, which contributes its recruitment to the key promoter regions of downstream genes and activates the gene expression [14, 15]. Further studies indicate that SMYD3 could be recruited to SMYD3 binding sites, which present in the downstream of the EZR promoter, causing local accumulation of SMYD3 and accompanied H3K4Me3 at the EZR gene [16]. lncRNA EZR antisense RNA 1 (*EZR-AS1*) is 362 bp in length and is located on chromosome 6q25.3. The expression of *EZR-AS1* may promote cell migration and mediate cancer cell differentiation [17]. Despite these discoveries, the role of lncRNA *EZR-AS1* in noncancerous pathologies, particularly cardiovascular diseases, remains unclear.

Therefore, the aim of the present study was to determine the function of *EZR-AS1* in CHD, by assessing the proliferation, migration, and apoptotic rates of human venous endothelial cells (HUVECs) following the knockdown and overexpression of lncRNA *EZR-AS1*.

## 2. Materials and Methods

### 2.1. Clinical Blood Samples

In the present study, 35 patients (24 men and 11 women; 50-75 years of age) were recruited, who had been angiographically diagnosed with CHD at the Guizhou Provincial People's Hospital (Guiyang, Guizhou, China) between June 2018 and August 2018. In addition, 38 individuals without CHD (22 men and 16 women; 50-75 years of age) were enrolled as controls. The inclusion criteria included patients with stable angina pectoris exhibiting ≥1 major coronary artery with >80% stenosis. The exclusion criteria included patients with (i) unstable angina or myocardial infarction; (ii) CHD complicated with other organic heart diseases; and (iii) CHD combined with severe liver disease, kidney diseases, familial hypercholesterolemia, malignant tumors, or inflammatory diseases. All blood samples were immediately stored at 4°C, and RNA was extracted on the same day. The study protocol was approved by the Human Ethics Committee Review Board of the Guizhou Provincial People's Hospital, and oral informed consent was obtained from each patient (Ethics approval No. (2019)068).

### 2.2. Cell Culture and Transfection

HUVECs were purchased from the Xiangya Cell Bank of Central South University. The cells were cultured in Roswell Park Memorial Institute (RPMI) 1640 medium (HyClone; GE Healthcare Life Sciences) supplemented with 10% fetal bovine serum (Biological Industries) and 1% penicillin/streptomycin (Beijing Solarbio Science & Technology Co., Ltd.), at 37°C (5% CO_2_). Cells in the logarithmic phase were harvested for further experimentation. The HUVECs were transiently transfected with negative control small interfering (si) RNA (si-control) or si-*EZR-AS1*; the pcDNA-control, pcDNA-*EZR-AS1*, pcDNA-EZR, or pcDNA-SMYD3; si-*EZR-AS1*+pcDNA-EZR; or si-*EZR-AS1*+pcDNA-SMYD3 as appropriate. Transfection was carried out with 50 nM siRNA or 1.6 *μ*g/ml pcDNA using Lipofectamine 2000 (Invitrogen; Thermo Fisher Scientific, Inc.) according to the manufacturer's instructions. When simultaneously transfecting siRNA and pcDNA, 50 nM siRNA and 1.6 *μ*g/ml pcDNA were also used. The sequences of the siRNAs are as follows: si-*EZR-AS1*, 5′-UAUUUUCCAAAUCUUUUCCTT-3′; si-SMYD3, 5′-UCACAGCUGUGACCCCAACTT-3′; and si-EZR, 5′-GCUCAAAGAUAAUGCUAUGTT-3′.

### 2.3. Cell Counting Kit-8 (CCK-8) Assay

HUVECs were seeded into 96-well culture plates at a density of 3 × 10^3^ cells/well. The following day, the HUVECs were transfected with the corresponding siRNAs or overexpression pcDNA for 24, 48, and 72 h. Cell viability was assessed using the CCK-8 assay (Dojindo Molecular Technologies, Inc.) according to the manufacturer's protocol; 10 *μ*l CCK-8 solution was added to each well, and the plates were incubated for 2 h. The absorbance was then measured at 450 nm using a plate reader.

### 2.4. Wound Healing Assay

HUVECs were plated in 6-well cell culture plates, and 48 h after transfection, the cell monolayers were scratched with a 200 *μ*l plastic pipette tip. After washing twice with phosphate-buffered saline (PBS), the cells were incubated for 24 h in low-serum medium. The wound closure distance was then determined using an inverted microscope (Olympus Corporation) at ×100 magnification, and the area of closure between the 0 and 24 h time points was calculated.

### 2.5. Transwell Assay

The cells (5 × 10^4^/plate) were resuspended in 0.1 ml serum-free culture medium and seeded into the upper chamber of a Transwell insert (8 *μ*m pore size, 24-well; Corning Inc.). In the lower chamber, 0.8 ml complete culture medium was added as a chemoattractant. Following incubation at 37°C for 48 h, the cells that had migrated to the lower surface were fixed with methanol for 20 min and then stained with 0.1% crystal violet solution for a further 20 min. Finally, the stained cells were counted under an inverted microscope (Olympus Corporation) at ×100 magnification.

### 2.6. Flow Cytometry

HUVECs were cultured in 6-well plates to 70-90% confluence. The cells were then digested using trypsin and collected by centrifugation (1000 rpm at 4°C for 5 min). Following two washes with precooled PBS, the cells were collected and resuspended in 500 *μ*l binding buffer (1X); 5 *μ*l Annexin V-APC and 5 *μ*l 7-AAD (Nanjing KeyGen Biotech Co., Ltd.) were added, and the cells were incubated at room temperature in the dark for 15 min. Apoptotic cells were then analyzed using a FACScan flow cytometer (BD Biosciences).

### 2.7. Reverse Transcription-Quantitative PCR (RT-qPCR)

The extraction of RNA from the peripheral blood samples was conducted using Total RNA Extraction Kit of blood according to the manufacturer's instructions (BioTeke Corporation). Total RNA was isolated from cells using TRIzol® reagent (Invitrogen; Thermo Fisher Scientific, Inc.). The RNA was then reverse transcribed into first-strand cDNA using the PrimeScript RT Reagent Kit (Takara Bio, Inc.), and the expression levels of the target RNA were determined by RT-qPCR analysis using the Two-Step SYBR PrimeScript RT-PCR Kit (Takara Bio, Inc.) on an Illumina P05775 system (Illumina, Inc.). Glyceraldehyde 3-phosphate dehydrogenase (GAPDH) was used as the normalization control, and the thermocycling conditions were as follows: one cycle at 95°C for 1 min; 40 cycles at 95°C for 15 sec and 60°C for 1 min; and one cycle at 95°C for 15 sec, 60°C for 1 min, and 95°C for 15 sec. The primers were supplied by Sangon Biotech Co., Ltd., and the sequences are as follows: lncRNA *EZR-AS1*, forward 5′-CCAATGAAGCCTCTCCCGTC-3′ and reverse 5′-GGGAGATAACAGGCCCTGAC-3′; EZR, forward 5′-GTGTGGTACTTTGGCCTCCA-3′ and reverse 5′-AACTTGGCCCGGAACTTGAA-3′; SMYD3, forward 5′-CCCTCGGGCGTACGTG-3′ and reverse 5′-CTTGGCGGTTGCGAACTTTT-3′; and GAPDH, forward 5′-AGCCACATCGCTCAGACAC-3′ and reverse 5′-GCCCAATACGACCAAATCC-3′. Relative gene expression was calculated using the 2^-*ΔΔ*Cq^ method [18].

### 2.8. Western Blotting

HUVECs were seeded into 6-well cell culture plates and incubated at 37°C overnight. The following day, the cells were transfected with the corresponding siRNAs or overexpression pcDNA. Radioimmunoprecipitation assay buffer (Beijing Solarbio Science & Technology Co., Ltd.) supplemented with complete protease inhibitor cocktail (Roche Molecular Diagnostics) was used to lyse the cells, and the protein concentration was quantified using a bicinchoninic acid protein assay kit (Beyotime Institute of Biotechnology). 40 *μ*g of protein extract was separated by SDS-PAGE (using 10 or 12% gels) and transferred to polyvinylidene fluoride membranes (EMD Millipore). The membranes were blocked for 2 h with 5% nonfat milk (at room temperature) and then incubated with primary antibodies at 4°C overnight. The membranes were subsequently washed with TBST (0.1% Tween 20) and then incubated with the secondary antibody (cat. no. bs-40296G-HRP; 1 : 5,000; BIOSS) for 1.5 h at room temperature. An enhanced chemiluminescence kit (EMD Millipore) was used to visualize the blots, and the protein bands were quantified using ImageJ 180 software (National Institutes of Health). The primary antibodies used in the present study were as follows: anti-SMYD3 (cat. no. 12859; 1 : 1,000), anti-EZR (cat. no. 3145; 1 : 1,000), anti-phosphatase and tensin homolog (PTEN; cat. no. 9188; 1 : 1,000), anti-Bcl-2-associated X protein (Bax; cat. no. 5023; 1 : 1,000), anti-B cell lymphoma/lewkmia-2 (Bcl-2; cat. no. 4223; 1 : 1,000) (all Cell Signaling Technology, Inc.), anti-vascular endothelial growth factor (VEGF; cat. no. Sc-7269; 1 : 1,000; Santa Cruz Biotechnology), and anti-GAPDH (cat. no. AB-P-R001; 1 : 1,000; Goodhere, Inc.).

### 2.9. Statistical Analysis

Each experiment was repeated ≥3 times, and numerical data are presented as mean ± SD. The differences between the means were analyzed using Student's unpaired *t*-tests or one-way ANOVA where appropriate. All statistical analyses were performed using GraphPad 7.0 software (GraphPad Software, Inc.), and *P* < 0.05 was considered to indicate a statistically significant difference.

## 3. Results

### 3.1. Expression of lncRNA *EZR-AS1* and EZR in the Peripheral Blood

**Figure 1:**
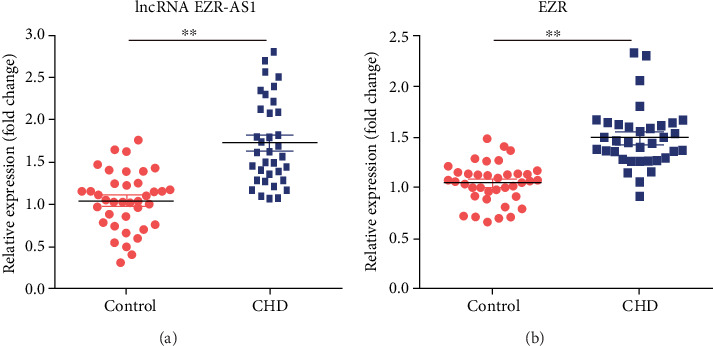
Expression of lncRNA *EZR-AS1* and EZR in peripheral blood. Reverse transcription-quantitative PCR was used to detect the expression levels of (a) lncRNA *EZR-AS1* and (b) EZR in the peripheral blood of control subjects and patients with CHD. All results were expressed as mean ± SD. ^∗∗^*P* < 0.01 vs. controls.

Initially, RT-qPCR was used to analyze the expression of *EZR-AS1* and EZR in the peripheral blood of 35 patients with CHD and 38 control subjects. The results revealed that *EZR-AS1* expression was markedly upregulated in patients with CHD, compared with the controls (Figure 1(a)). Similarly, EZR expression was also elevated in patients with CHD (Figure 1(b)).

### 3.2. Effects of lncRNA *EZR-AS1* on Proliferation, Migration, and Apoptosis in HUVECs

**Figure 2:**
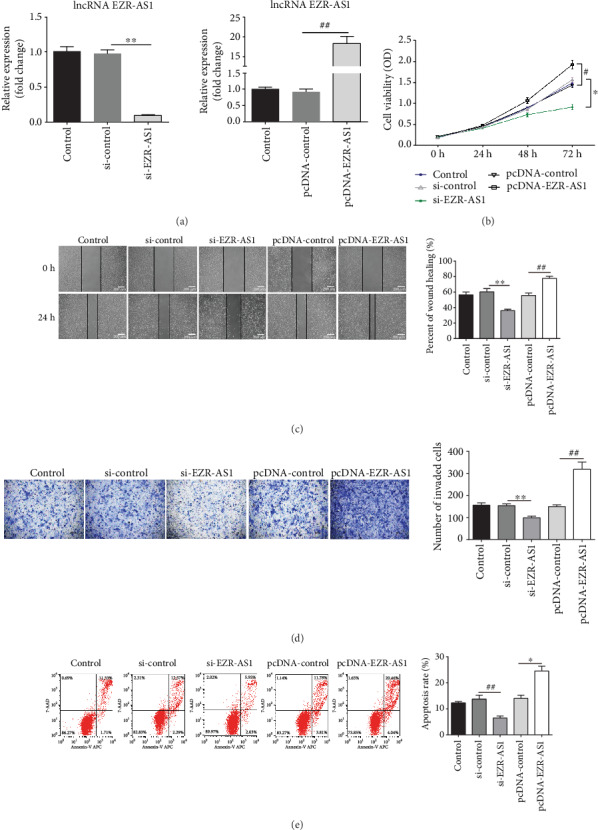
Effects of lncRNA *EZR-AS1* on human venous endothelial cell proliferation, migration, and apoptosis. Cells were transfected with (a) lncRNA si-*EZR-AS1* (knockdown) or si-control and with pcDNA-*EZR-AS1* (overexpression) or pcDNA-control. Untreated cells were used as a control. Posttransfection with si-*EZR-AS1* or pcDNA-*EZR-AS1*, (b) viability, (c, d) migratory ability, and (e) apoptotic rates of the cells were detected by CCK-8 assay, wound healing assay, Transwell assay, and flow cytometry, respectively. Wound healing was quantified, and the number of migratory cells was counted. Magnification, ×100 (migration). All results were expressed as mean ± SD. ^∗^*P* < 0.05 and ^∗∗^*P* < 0.01 vs. si-control; ^#^*P* < 0.05 and ^##^*P* < 0.01 vs. pcDNA-control.

To explore the functions of *EZR-AS1* in HUVECs, the cells were transfected with siRNA or overexpression pcDNA of EZR-AS1; transfection efficiency is illustrated in Figure 2(a). The results of the CCK-8 assay showed that *EZR-AS1* knockdown reduced cell viability (Figure 2(b)). Additionally, si-*EZR-AS1* suppressed the migration (Figures 2(c) and 2(d)) and apoptosis (Figure 2(e)) of HUVECs. Overexpression of *EZR-AS1* resulted in the reverse effect.

### 3.3. Effects of lncRNA *EZR-AS1* on the Expression Levels of Apoptosis-Related Proteins in HUVECs

**Figure 3:**
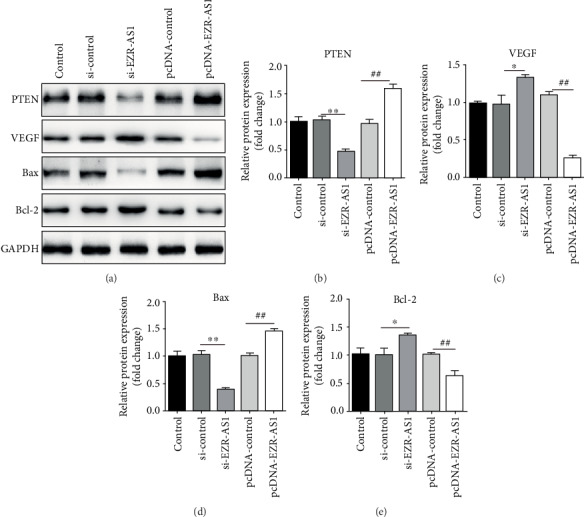
Effects of lncRNA *EZR-AS1* on apoptosis-related protein levels in human venous endothelial cells. (a) Western blotting was used 48 h after transfection to detect the protein expression levels of (b) PTEN, (c) VEGF, (d) Bax, and (e) Bcl-2. All results were expressed as mean ± SD. ^∗^*P* < 0.05 and ^∗∗^*P* < 0.01 vs. si-control; ^#^*P* < 0.05 and ^##^*P* < 0.01 vs. pcDNA-control.

To determine whether lncRNA *EZR-AS1* mediated apoptosis, Western blotting was used to detect the expression levels of apoptosis-related proteins, including PTEN, VEGF, Bax, and Bcl-2. *EZR-AS1* knockdown increased the expression levels of VEGF and Bcl-2, while decreasing those of PTEN and Bax. The opposite effect was observed when *EZR-AS1* was overexpressed (Figures 3(a)–3(e)). These data indicate that downregulating the expression of lncRNA *EZR-AS1* inhibits apoptosis in HUVECs.

### 3.4. lncRNA *EZR*-*AS1* Positively Regulates SMYD3 and EZR Expression

**Figure 4:**
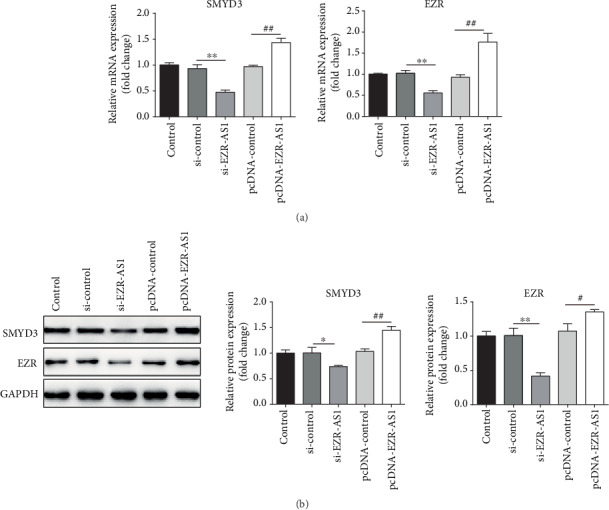
SMYD3 and EZR are positively regulated by lncRNA *EZR-AS1*. The (a) mRNA and (b) protein expression levels of SMYD3 and EZR were detected in cells transfected with si-*EZR-AS1* or pcDNA-*EZR-AS1*. All results were expressed as mean ± SD. ^∗^*P* < 0.05 and ^∗∗^*P* < 0.01 vs. si-control; ^#^*P* < 0.05 and ^##^*P* < 0.01 vs. pcDNA-control.

The expression levels of SMYD3 and EZR were analyzed via lncRNA *EZR-AS1* knockdown or overexpression. The results showed that the downregulation of *EZR-AS1* decreased the expression of SMYD3 and EZR, and the opposite effect was observed when *EZR-AS1* was overexpressed. These results indicate that lncRNA *EZR-AS1* positively regulates the expression of SMYD3 and EZR (Figures 4(a) and 4(b)).

### 3.5. Overexpression of EZR Reverses the Effects of *EZR-AS1* Knockdown on the Biological Properties of HUVECs

**Figure 5:**
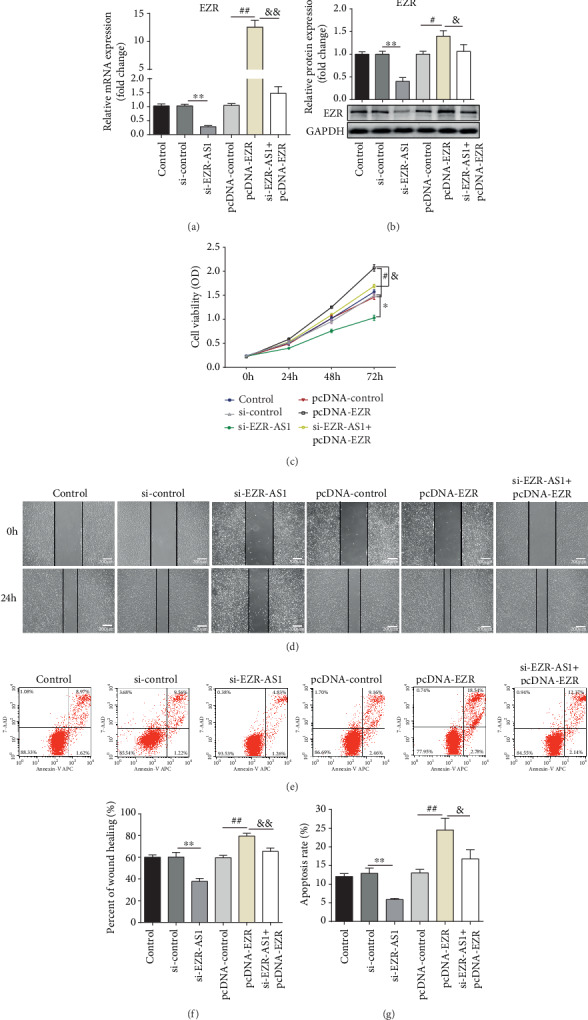
Effects of pcDNA-EZR on human venous endothelial cells. After transfected with pcDNA-EZR or si-*EZR-AS1*+pcDNA-EZR, the (a) mRNA and (b) protein expression levels of EZR were determined using reverse transcription-quantitative PCR and Western blotting. The (c) viability, (d, f) migratory ability, and (e, g) apoptosis of cells were detected using the CCK-8 assay, wound healing assay, and flow cytometry, respectively. Wound healing was quantified. Magnification, ×100 (migration). All results were expressed as mean ± SD. ^∗^*P* < 0.05 and ^∗∗^*P* < 0.01 vs. si-control. ^#^*P* < 0.05 and ^##^*P* < 0.01 vs. pcDNA-control. ^&^*P* < 0.05 and ^&&^*P* < 0.01 vs. pcDNA-EZR.

To further explore whether EZR was involved in the effects of si-*EZR-AS1*, HUVECs were transfected with an EZR overexpression pcDNA. RT-qPCR and Western blotting confirmed that the EZR expression level was significantly decreased in cells transfected with both si-*EZR-AS1* and pcDNA-EZR (Figures 5(a) and 5(b)), compared with those transfected with pcDNA-EZR alone. Proliferation, migration, and apoptotic capacity were evaluated after transfection; the results showed that the overexpression of EZR significantly reversed the effects of si-*EZR-AS1* on cell proliferation, migration, and apoptosis (Figures 5(c)–5(g)).

### 3.6. Overexpression of SMYD3 Reverses the Effect of si-*EZR-AS1* on the Biological Properties of HUVECs

**Figure 6:**
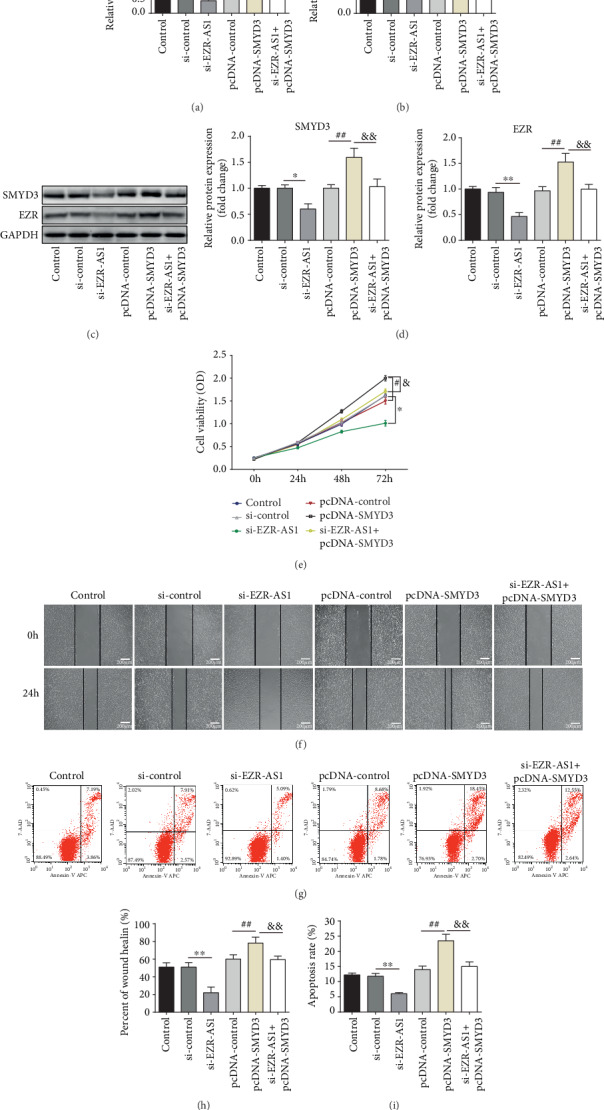
*EZR-AS1* mediates EZR expression via SMYD3. After transfected with pcDNA-SMYD3 or si-*EZR-AS1*+pcDNA-SMYD3, the (a, b) mRNA and (c, d) protein expression levels of SMYD3 and EZR were determined by reverse transcription-quantitative PCR and Western blotting. (e) Cell viability was detected by CCK-8 assay. (f, h) Migratory ability was detected by wound healing assay. (g, i) Apoptosis was detected by flow cytometry. Wound healing was quantified. Magnification, ×100 (migration). All results were expressed as mean ± SD. ^∗^*P* < 0.05 and ^∗∗^*P* < 0.01 vs. si-control. ^#^*P* < 0.05 and ^##^*P* < 0.01 vs. pcDNA-control.^&^*P* < 0.05 and ^&&^*P* < 0.01 vs. pcDNA-SMYD3.

Finally, to investigate the involvement of SMYD3 in the effects of lncRNA *EZR-AS1*, HUVECs were transfected with pcDNA-SMYD3 or si-*EZR-AS1*+pcDNA-SMYD3. The results indicated that SMYD3 overexpression could reverse the effects of *EZR-AS1* knockdown, resulting in the upregulation of SMYD3 and EZR (Figures 6(a)–6(d)). At the same time, the upregulation of SMYD3 reversed the proliferation, migration, and apoptotic ability of si-*EZR-AS1*-transfected cells (Figures 6(e)–6(i)).

## 4. Discussion

Cardiovascular diseases, particularly CHD and stroke, remain the leading global causes of death, and the lifetime risk of CHD is 67% in individuals > 55 years of age [19–21]. Therefore, new strategies for the treatment of CHD are urgently required. lncRNAs have been associated with the treatment of various cardiovascular diseases, including CHD [22, 23]; thus, defining the functions of lncRNAs may aid in the development of novel diagnostic and therapeutic targets for CHD.

Generally speaking, CHD is diagnosed when stenosis of the coronary artery reaches >50%. In the study, RT-qPCR was used to analyze the expression of *EZR-AS1* in the peripheral blood of patients with severe CHD (with stenosis > 80%) and controls. The results showed that *EZR-AS1* was robustly upregulated in patients with severe CHD. To the best of our knowledge, this was the first study to have evaluated the role of lncRNA *EZR-AS1* in CHD. Based on these findings, the functions of lncRNA *EZR-AS1* were further assessed through *in vitro* experimentation.

Vascular endothelial cells are important components of the vascular wall. The abnormal proliferation and migration of vascular endothelial cells are important pathophysiological processes in diseases such as CHD, hypertension, and restenosis after percutaneous coronary intervention [24–26]. In the present study, the role of *EZR-AS1* was evaluated in HUVECs by assessing cell proliferation, migration, and apoptosis; this was achieved by transfection with si-*EZR-AS1* or an overexpression pcDNA. The results revealed that the downregulation of *EZR-AS1* significantly inhibited HUVECs proliferation, migration, and apoptosis; the opposite effects were observed following *EZR-AS1* overexpression. These findings suggest that lncRNA *EZR-AS1* influences the malignant behaviors of HUVECs, providing a basis for its therapeutic application in CHD.

EZR, encoded by Vil2, was identified as the first member of the ERM family. EZR is upregulated in a number of diseases, such as breast and cervical cancer, and overexpression of the EZR gene may enhance the metastatic capacity of tumors [27, 28]. Although the function of EZR has been extensively studied, the transcriptional regulation of the EZR gene is still poorly understood. To the best of our knowledge, the role of EZR in the progression of CHD remains unclear. In the current study, EZR was revealed to be upregulated in patients with CHD, and as predicted, increased levels of EZR promoted the proliferation and migration of HUVECs. Similar to the effects of the lncRNAs *BDNF-AS* and *NEXN-AS1* on target gene expression [29, 30], knocking down *EZR-AS1* decreased the expression level of EZR. Additionally, EZR overexpression reversed the effects of *EZR-AS1* knockdown, indicating that EZR is involved in regulating the biological behaviors of HUVECs. Furthermore, siRNA suppressed the expression of *EZR-AS1* while simultaneously raised that of SMYD3, which could reverse the downregulation of EZR mediated by the si-*EZR-AS1*, which suggested that *EZR-AS1* is most likely to regulate EZR expression through SMYD3 signaling in HUVECs; more experiments such as RNA-binding protein immunoprecipitation (RIP) and chromatin immunoprecipitation (ChIP) should be done in our next work to further confirm it.

## 5. Conclusion

In conclusion, and to the best of our knowledge, the present study is the first to report that lncRNA *EZR-AS1* is upregulated in severe CHD and that the downregulation of *EZR-AS1* inhibits the proliferation, migration, and apoptosis of HUVECs via SMYD3. These data facilitate a deeper understanding of the molecular mechanisms of lncRNA *EZR-AS1* in CHD. Therefore, lncRNA *EZR-AS1* may be a potential biomarker for the diagnosis and treatment of severe CHD.

## Data Availability

The analyzed data sets used and/or analysed during the present study are available from the corresponding author on reasonable request.
